# Simulating the Effects of Partial Neural Conduction Delays in the Visual Evoked Potential

**DOI:** 10.1167/tvst.13.2.18

**Published:** 2024-02-22

**Authors:** Enyam K. A. Morny, Julia Haldina, Sven P. Heinrich

**Affiliations:** 1 Eye Center, Medical Center – University of Freiburg, Freiburg, Germany; 2 Faculty of Medicine, University of Freiburg, Freiburg, Germany; 3 Department of Optometry and Vision Science, University of Cape Coast, Cape Coast, Ghana

**Keywords:** optic neuritis, visual evoked potential (VEP), peak time, conduction delay, Pulfrich effect, response superposition

## Abstract

**Purpose:**

The purpose of this study was to understand the double peaks or broadening of P100 observed in some cases of optic neuritis by inducing conduction delays in healthy eyes through stimulus luminance manipulation in analogy to the perceptual Pulfrich effect.

**Methods:**

Checkerboard pattern reversal visual evoked potentials (VEPs) with check sizes of 0.8 degrees, 0.4 degrees, and 0.2 degrees were recorded in healthy participants using two experiment variants. Variant (1) involved binocular stimulation with inter-ocular luminance difference achieved by a 1.8 neutral density (ND) filter, along with monocular control conditions. Variant (2) included monocular stimulation with hemifields having a luminance difference (half of monitor with ND filter), along with single-hemifield control conditions. In both variants, VEP curves under mixed stimulation were compared to synthesized VEPs computed from offline summation of curves from the relevant control conditions, followed by assessing P100 characteristics.

**Results:**

Despite considerable variability between participants, the binocular variant demonstrated marked differences between VEPs from mixed recordings and synthesized curves, whereas in the hemifield variant, agreement was strong. The anticipated double peak or broadened deflection pattern was observed to varying extents in participants, often contingent on check size, with nominal peak time frequently failing to indicate partial conduction delays.

**Conclusions:**

The present findings corroborate the hypothesis that nominal peak time does not always reflect conduction delays if only a subset of fiber bundles is affected. Peak shape might provide additional diagnostic evidence of a partial conduction delay.

**Translational Relevance:**

Enhancing the understanding of VEP waveform changes associated with partial conduction delays could offer diagnostic insights for optic neuritis.

## Introduction

The visual evoked potential (VEP) primarily represents the response of the visual cortex to stimuli presented in the central visual field.^1^ VEPs thus depend on functional integrity of central vision at all levels of the visual pathway from the eye up to the occipital cortex.^2^ This makes the VEP a very useful tool for understanding processes and assessing pathologies of the visual pathway.

For this reason, a common clinical application of VEPs is the diagnosis of optic neuritis,^3^^–^^6^ which is an acute inflammation of the optic nerve. In this regard, VEP to a pattern reversal checkerboard stimulus is most used and it consists of a negative peak around 75 ms (N75), a positive component at approximately 100 ms (P100), and a later negative peak at around 135 ms (N135).^2^ Generally, axonal degeneration produces a reduction in VEP amplitude and changes in the morphology of the VEP responses. In contrast, the demyelination of axons in optic neuritis, resulting from the inflammation of the optic nerve, produces conduction delays in the optic nerve which commonly manifest in the VEP as a delayed P100. Notably, the delay in peak time is not fixed and in some cases of optic neuritis the delays are progressively shortened after the initial acute phase until the peak time of the affected eye returns to normal limits within 2 to 3 years.^4^ This is attributed to remyelination of the optic nerve axons, although this does not correspond to a return to normal function of the initially affected axons.^4^ Nevertheless, demyelination diseases also lead to conduction block, where there is complete signal transmission failure of some or all axons; or temporal dispersion, where nerve fibers conduct signals at different velocities.^7^^,^^8^ Both mechanisms can lead to diminished amplitudes.^7^^,^^8^ In conduction block, this occurs because nerve fibers that are blocked do not contribute to the VEP. In temporal dispersion, this occurs because asynchronous signals combine in a destructive manner due to phase cancelation.^7^^,^^9^

**Figure 1.**
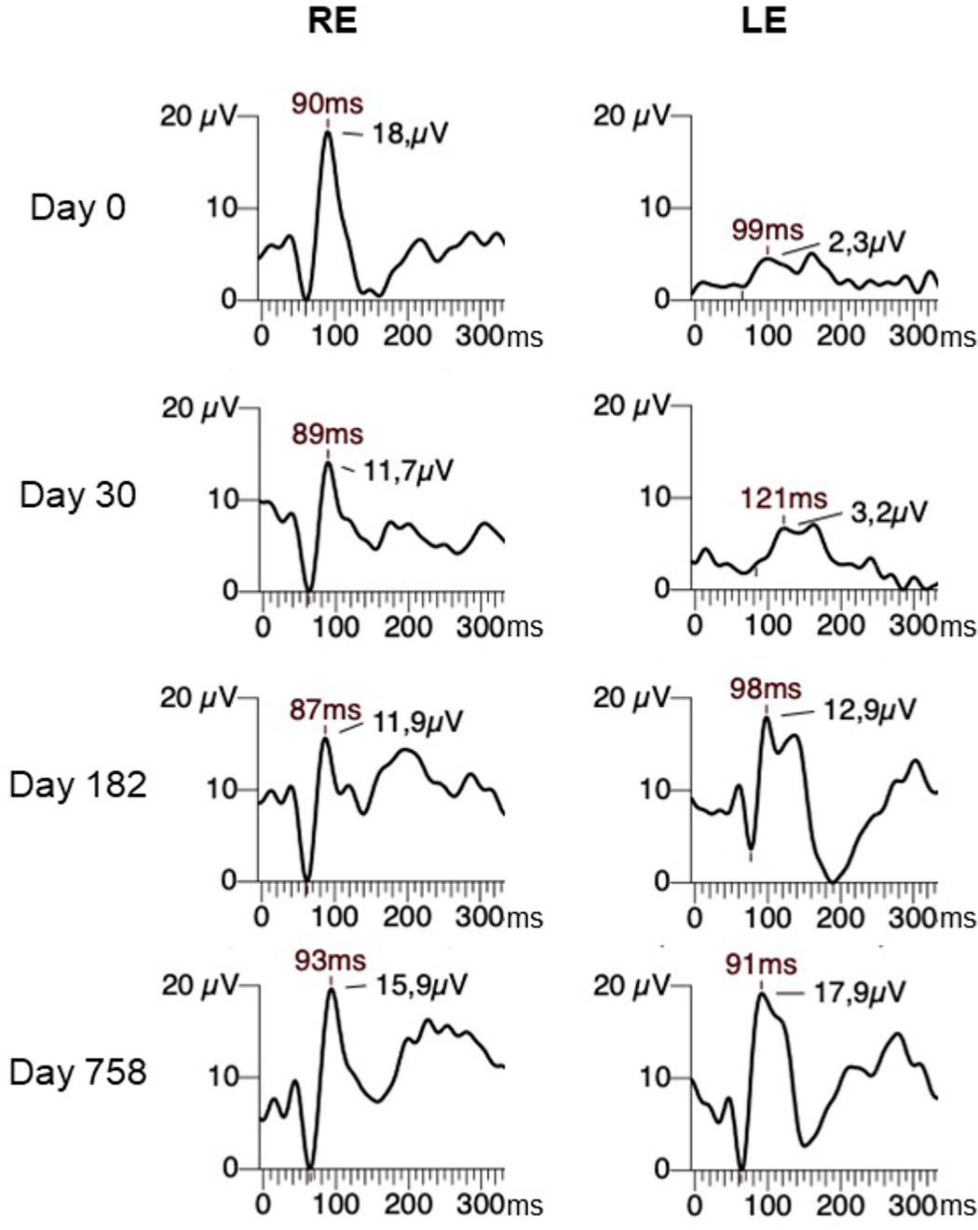
VEP traces of a case of optic neuritis followed up for 2 years, with normal right eye and affected left eye. *Left*: VEP is almost flat at day 0, double peaks appear at day 30, persist through day 182, and disappear by day 758. Credit: Treatment of Optic Neuritis with Erythropoietin Study.

Observations of the VEP traces from a cohort of acute optic neuritis cases (patients included in the TONE clinical trial^10^^,^^11^ at the local study site) which were followed up over a course of 2 years, showed the emergence of a double peak or broadening of the P100 in some of the cases recorded at the initial measurement (Fig. 1). This often persisted through the 3 and 6 month follow-up VEPs but disappeared in the 2-year follow-up VEPs. This double peaking or broadening of the P100 often confounded amplitude and peak time measurement of the P100. We hypothesized that these curve shape alterations originated from conduction delays that affected only some of the fiber bundles of the optic nerve. The emergence of the double peak may reflect the asynchrony of signal transmission between the healthy fibers and unhealthy fibers, and the subsequent disappearance of the double peak at later stages may reflect re-synchronization due to an increase in the proportion of recovered (remyelinated) fibers. Our clinical experience suggests that double peaks predominantly occur in patients with demyelinating diseases, although there are occasionally cases with no apparent underlying pathology.

Partial conduction delays in the visual system can be simulated using neutral density filters.^12^^–^^14^ The effect arises from the fact that dimming a stimulus with a neutral density filter slows down retinal processing in the eye with filter^12^ thereby increasing signal conduction time between that eye and the cortex.^13^^–^^15^

By simulating these delays (using dimming-induced effects as an experimental tool without replicating the actual pathophysiological processes associated with optic neuritis), this study aimed to advance the understanding of the effect of conduction delays on the waveform of the VEP and to potentially facilitate the interpretation of VEP findings that involve double peaks or peak broadening.

This experimental approach is known from the perceptual Pulfrich effect.^13^^–^^15^ This is a visual stereo illusion where lateral motion is perceived as having a depth component. It can be observed when a swinging pendulum bob is viewed through a neutral density filter in front of one eye. Although the bob is moving in a fronto-parallel plane, the path seems elliptical because of the interocular signal latency difference between the filtered and unfiltered eye, which simultaneously establishes a seeming spatial disparity in which the object appears to be in two different locations with the image from the filtered eye appearing to lag behind the image from the unfiltered eye.^13^^,^^16^^,^^17^ This disparity cue stimulates disparity tuned neurons to give rise to the perception of depth, that is, the elliptical pathway Furthermore, a spontaneous Pulfrich effect is a common symptom reported by people with unilateral optic neuritis.^13^^,^^16^^,^^17^ To correct this, neutral density filters of different strengths are placed in front of the unaffected eye until the effect is eliminated.^13^^,^^17^^,^^18^ The forementioned cases together suggest that an interocular signal latency difference may, in itself, act as an independent cue for depth perception.

## Materials and Methods

### Participants

VEPs were recorded from 18 visually healthy participants with best corrected visual acuity of -0.1 logMAR or better. However, data for three participants were excluded for the following reasons: incomplete data due to fatigue and inability of the participant to return to complete the recordings (*n* = 2) and excessive artefacts due to excessive blinking (*n* = 1). The study belonged to a series of experiments approved by the local institutional review board and followed the tenets of the Declaration of Helsinki. All participants provided their written consent after receiving a participant information sheet and having the opportunity to ask questions.

### Stimulation

Following the principles laid down in the respective International Society for Clinical Electrophysiology of Vision (ISCEV) standards,^19^ pattern reversal checkerboard stimuli with check sizes 0.8 degrees, 0.4 degrees, and 0.2 degrees, a contrast of 98% and a mean luminance of 360 cd/m^2^ were used. Reversal rate was 2 reversals per second. The distance between the eye and the monitor was 114 cm and the total extent of the stimulus was 19 degrees × 15 degrees (length × height).

The VEP was recorded from the Oz position, referenced to Fpz^20^ and with ground connected to one ear lobe. Signals were band-passed at 1 to 100 Hz, amplified 50,000-fold, and sampled at 1 kHz. Artifacts were rejected based on a ± 130 µV threshold criterion. Eighty artifact-free trials per check size were recorded and averaged. For display and further analysis, a digital non-causal 45-Hz low-pass filter was applied to the traces. Participants wore their best spectacle correction with appropriate near addition and recordings were done with natural pupils. To ensure subject alertness, fixation, and accommodation, participants read out numbers (1 to 9) which flashed in pseudo-random order in the middle of the fixation target (diameter = 0.5 degrees).

**Figure 2.**
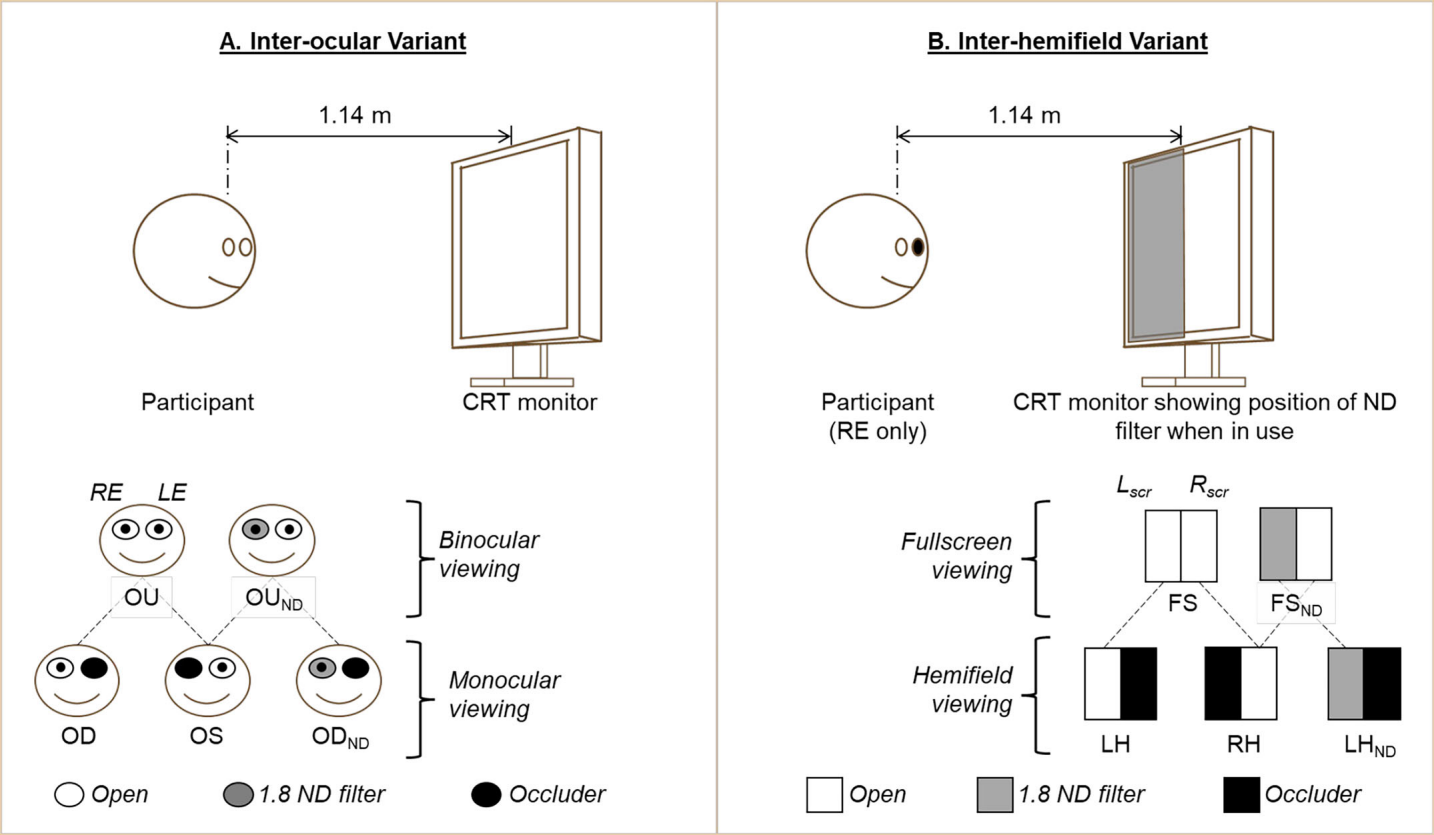
Participant and stimulus set up for VEP recording. Two variants of the experiment were recorded. (**A**) Interocular variant following the classical induction of the Pulfrich effect and (**B**) inter-hemifield variant following a modified Pulfrich-like effect for monocular induction. ND – neutral density filter; RE – right eye; LE – left eye; OU – VEP with both eyes open; OU_ND_ – VEP with both eyes open but with 1.8 ND in front of right eye; OD – VEP from right eye only; OS – VEP from left eye only; OD_ND_ – VEP from right eye only but with 1.8 ND in front of it; Lscr – Left half of screen; Rscr – right half of screen; FS – VEP from full screen (also the same as OD in panel **A**); FS_ND_ – VEP from full screen with 1.8 ND in front of left half of screen; LH – VEP from left hemifield; RH – VEP from right hemifield; LH_ND_ – VEP from left hemifield with 1.8 ND covering it.

Two variants of the experiments were conducted (Fig. 2). The first variant, henceforth called the interocular variant, followed the classical induction of the Pulfrich Effect (see Fig. 2A). This was used to validate the approach in the context of VEP recordings. VEPs were recorded with both eyes open but with a neutral density (1.8 ND) filter in front of the right eye. A control condition without the ND filter was also recorded (i.e. both eyes open without the filter). VEPs were then recorded monocularly from each eye to provide the individual parts that were later summed up offline to obtain synthesized binocular VEPs for the test and control conditions of the interocular variant. As such, VEPs were recorded monocularly from the right eye (OD), left eye (OS), and right eye with 1.8 ND (OD_ND_).

In the second variant, henceforth called the inter-hemifield variant, the above Pulfrich-like version was modified for monocular induction. This more closely resembled the situation when a subset of optic nerve fibers is affected in unilateral optic neuritis. VEPs were recorded from only the right eye, with a 1.8 ND covering the left half of the monitor (see Fig. 2B). The control condition in this case was the same eye viewing the full screen without any filters. Hemifield VEPs were then recorded for the right eye and later summed up offline to obtain synthesized the responses obtained to stimulation of both hemifields. The hemifield VEPs were recorded from the left hemifield (LH), right hemifield (RH) and LH with 1.8 ND filter (LH_ND_). For each hemifield recording, the opposite hemifield was occluded with an opaque card covering half of the monitor. For six of the participants, pattern electroretinograms (PERGs) were recorded simultaneously using DTL electrodes to assess retinal contributions to the effect. Left and right instead of upper and lower hemifields were chosen to avoid effects of polarity inversion in the VEP.^21^

### Statistical Analysis

The amplitude of the N75 was measured from baseline to the trough of the N75, P100 amplitude was measured from the trough of the N75 to the peak of the P100, and N135 amplitude was measured from the peak of the P100 to the trough of N135. Peak time was measured from stimulus onset to the trough or peak of the various components. All peaks and troughs were determined objectively using Microsoft Excel to find the maximum or minimum voltage within a fixed time window. A time window of 75 to 105 ms was used for all conditions, except the OD_ND_ condition where a 90 to 130 ms window was used to account for the delays in peak time.

Statistical comparisons (paired-sample *t*-tests) for amplitudes and peak times were limited to the P100 component for simplicity. Comparisons between the waveform shape of different test conditions were made qualitatively and quantitatively. The qualitative assessments were made by overlapping two traces of interest and judging how closely they matched with each other on a three-point scale of good, moderate, and poor. Quantitative assessments were made by calculating a correlation coefficient (R^2^) for two traces within a reference window. The start and end points of the reference window were defined respectively by the peak times of the N75 and N135 of the control condition. The quantitative comparison was limited to the shape of the P100.

## Results

Participants were aged between 18 and 38 years (mean = 25.3 ± 5.9 years). The results are presented in two main sections. The first part presents the results from the 15 participants and are limited to the VEPs recorded with the 0.8 check size for brevity. The second later section presents the results of the grand mean traces of the VEPs from the three check sizes.

### Section 1: Individual VEPs

**Figure 3.**
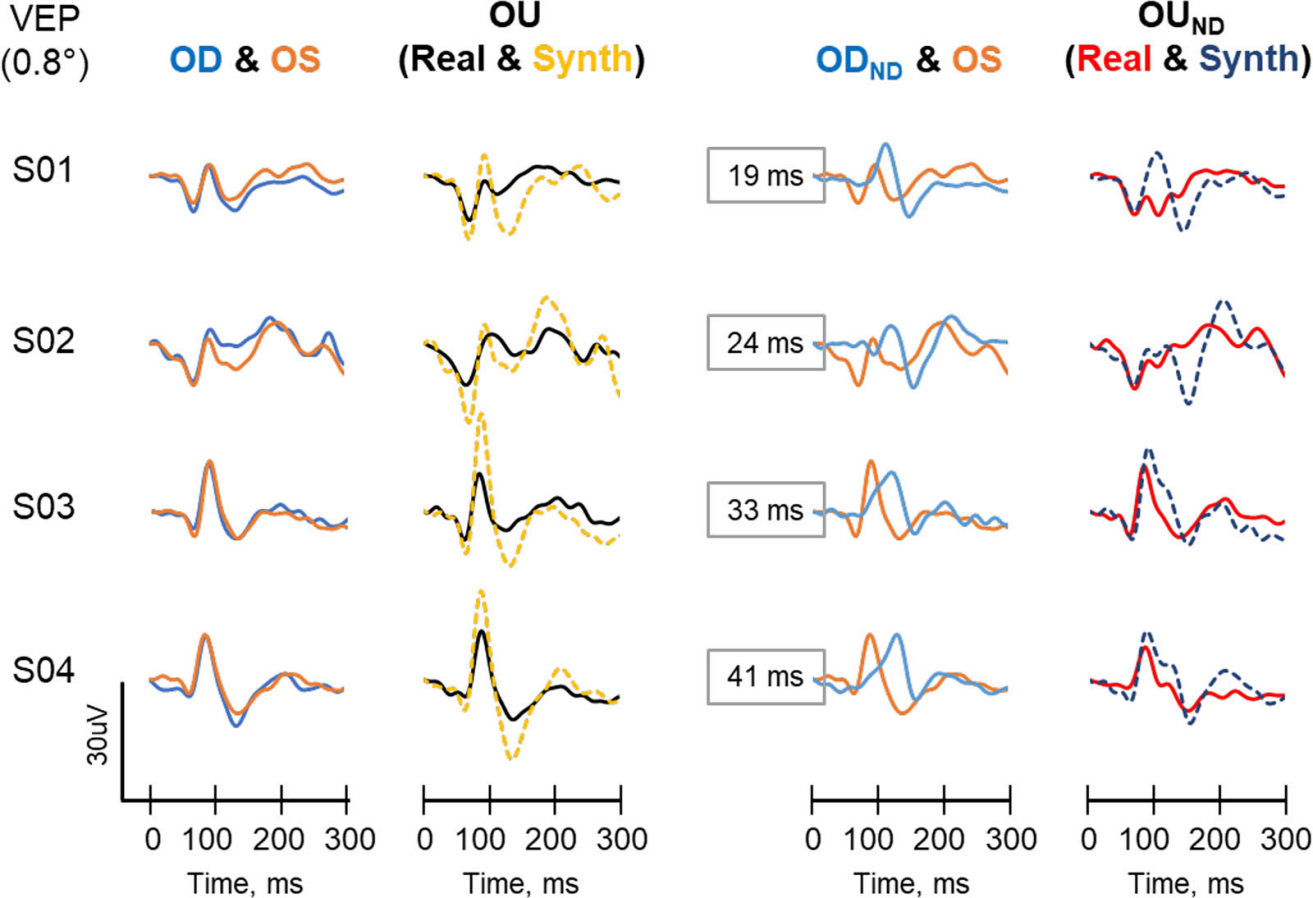
Example VEP traces of four participants for the interocular variant. *First column* shows the comparison of control VEP traces from the right (OD, *blue trace*) and left (OS, *orange trace*) eyes. *Second column* compares the real control binocular VEP trace (OU-Real, *black trace*) and its synthesized counterpart (OU-Sim, *yellow dash trace*). *Third column* shows the comparison between the VEP of the right eye with a 1.8 ND in front of it (OD_ND_) and the unfiltered left eye (OS). *Fourth column* compares the binocular VEP recorded with both eyes open but with a 1.8 ND in front of the right eye (OU_ND_, *red trace*), and its synthesized counterpart (*blue dash trace*). The traces are arranged vertically in order of increasing time difference between the P100 of the OD_ND_ and OS. The time differences are shown as numbers in the boxes in the *third column*.

**Figure 4.**
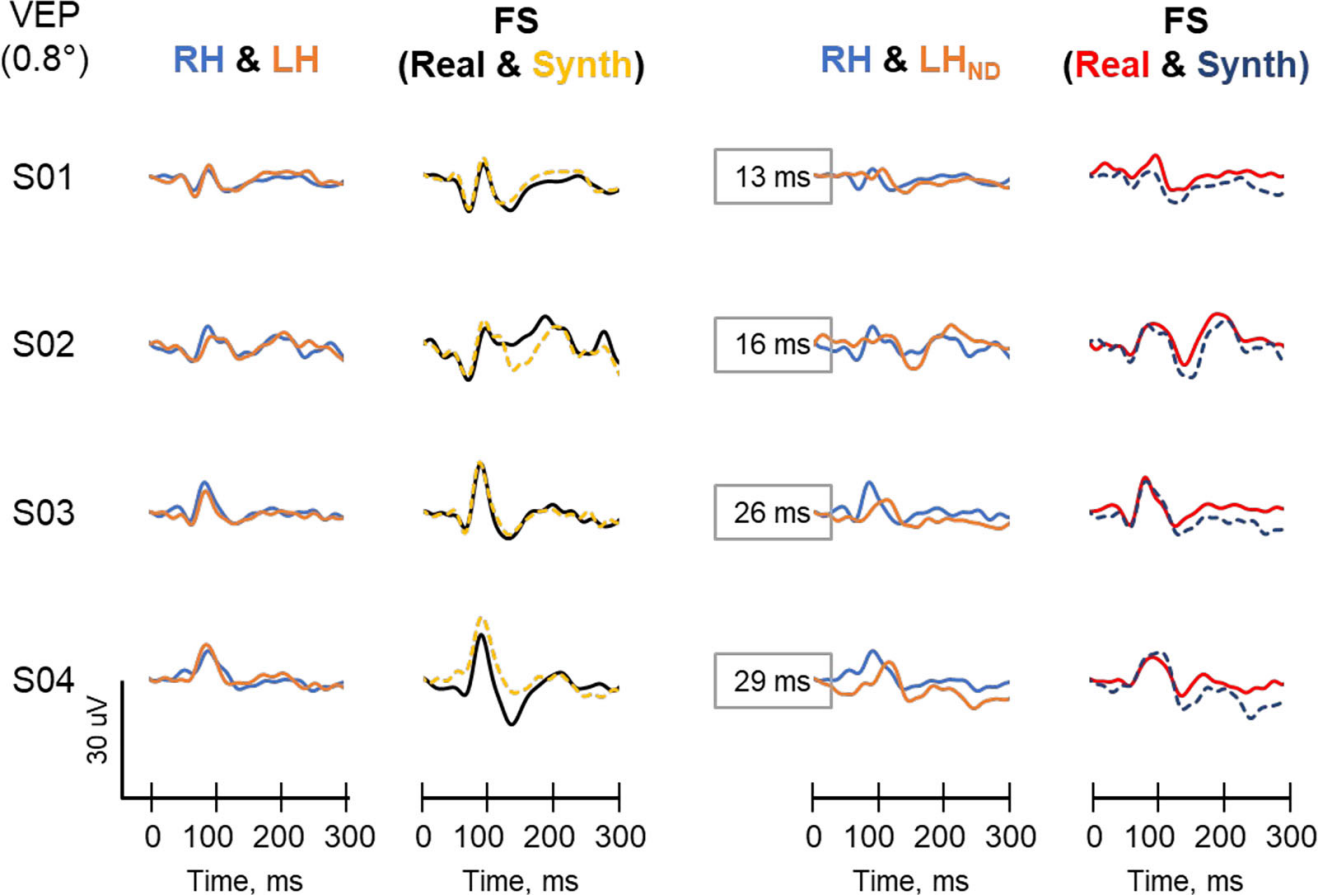
Example VEP traces of four participants for the inter-hemifield variant. Participants are the same as in Figure 3. First column shows the comparison of control traces from the right (RH, *blue trace*) and left (LH, *orange trace*) hemifields. *Second column* compares the real control full screen VEP trace (FS-Real, *black trace*) and its synthesized counterpart (FS-Sim, *yellow dash trace*). *Third column* shows the comparison between the VEP of the unfiltered right hemifield (RH) and the left 1.8 ND filtered hemifield (LH_ND_). *Fourth column* compares full screen VEP recorded with from the right eye but with a 1.8 ND in front of the left hemifield (FS_ND_, *red trace*), to its synthesized counterpart (*blue dash trace*). The traces are arranged vertically in order of increasing time difference between the P100 of the RH and LH_ND_. The time differences are shown as numbers in the *boxes* in the *third column*.

There were considerable variations in the waveforms between participants, which are exemplified by the traces from 4 participants in Figures 3 and 4 for the interocular and inter-hemifield variants, respectively. However, waveforms between the right and left eyes of each participant were very similar in amplitude, timing, and shape (see Fig. 3, column 1). There was no statistical difference between the right and left eyes with regard to mean P100 amplitude (OD = 13.7 ± 5.0 µV and OS = 12.8 ± 5.1 µV, *P* = 0.134), peak time (OD = 88.0 ± 4.5 ms and OS = 88.2 ± 3.9 ms, *P* = 0.745) and shape (mean R^2^ = 0.945 ± 0.064).

**Figure 5.**
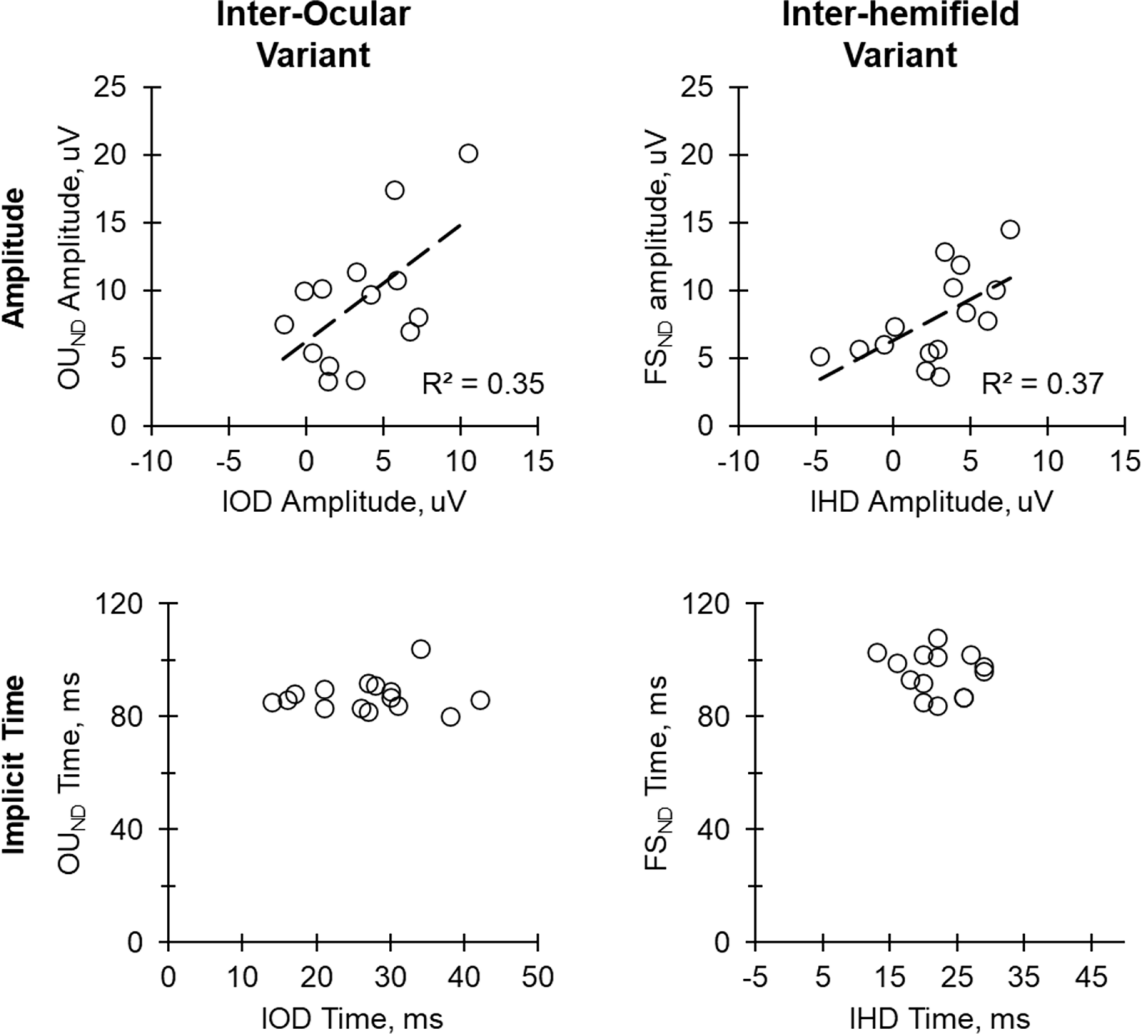
Left column: correlation between the interocular difference (IOD) in amplitude (between filtered [OD_ND_] and unfiltered [OD] eye) and the amplitude of test binocular VEP (OU_ND_), as well as the correlation between the IOD in implicit time and the implicit time of the OU_ND_. *Right column*: Correlation between the inter-hemifield difference (IHD) in amplitude (between filtered [LH_ND_] and unfiltered [LH] hemifield0 and the amplitude of test monocular VEP (FS_ND_) as well as correlation between the IHD in implicit time and the implicit time of the FS_ND_.

Comparisons between the real and synthesized binocular control VEP (OU; column 2), yielded a significant difference in the mean amplitude (real = 15.5 ± 5.8 µV and synthesized= 26.3 ± 9.9 µV, *P* < 0.001) but not in the peak time (real = 88.2 ± 5.5 ms and synthesized = OU: 88.2 ± 4.1 ms, *P* = 0.841). The waveform shapes were moderately similar (R^2^ = 0.761 ± 0.271). Interocular differences in peak time between the OD_ND_ and OS (represented by the numbers in the boxes in column 3) did not correlate significantly with the peak time of the OU_ND_ (Fig. 5). Again, a comparison of the real and synthesized binocular test conditions (OU_ND_) showed a significant difference in the amplitude (real = 9.0 ± 4.8 µV and synthesized = 15.0 ± 5.8 µV, *P* < 0.001) and peak time (real = 87.3 ± 5.8 ms and synthesized = 100.5 ± 10.5 ms, *P* = 0.001). The similarity between the waveforms was poor (R^2^ = 0.370 ± 0.305).

In the recordings for inter-hemifield variant, VEPs from the left (LH) and right (RH) hemifields were very similar (see Fig. 4, column 1). There was no significant difference between their mean amplitudes (RH = 7.4 ± 3.3 µV and LH = 7.7 ± 2.6 µV, *P* = 0.735) or peak times (RH = 87.1 ± 4.6 ms and LH = 88.0 ± 4.7 ms, *P* = 0.237). Furthermore, the real and synthesized control full screen (FS) VEP were very similar to each other. There was no significant difference in their amplitudes (real FS = 13.7 ± 5.0 µV and synthesized FS = 14.7 ± 5.2 µV, *P* = 0.735) or peak times (real FS = 88.0 ± 4.5 ms and synthesized FS = 88.9 ± 5.0 ms, *P* = 0.237). The real and synthesized waveforms in the test conditions (FS_ND_) were also similar with no significant difference in amplitude (real FS_ND_ = 7.9 ± 3.3 µV and synthesized FS_ND_ = 9.1 ± 4.3 µV) or peak time (real FS_ND_ = 94.7 ± 7.9 ms and synthesized FS_ND_ = 94.7 ± 8.9 ms). As was the case in the interocular variant, inter-hemifield differences between the RH and the LH_ND_ did not correlate significantly with the peak time of the FS_ND_ (see Fig. 5).

### Section 2: Grand Mean VEPs

**Figure 6.**
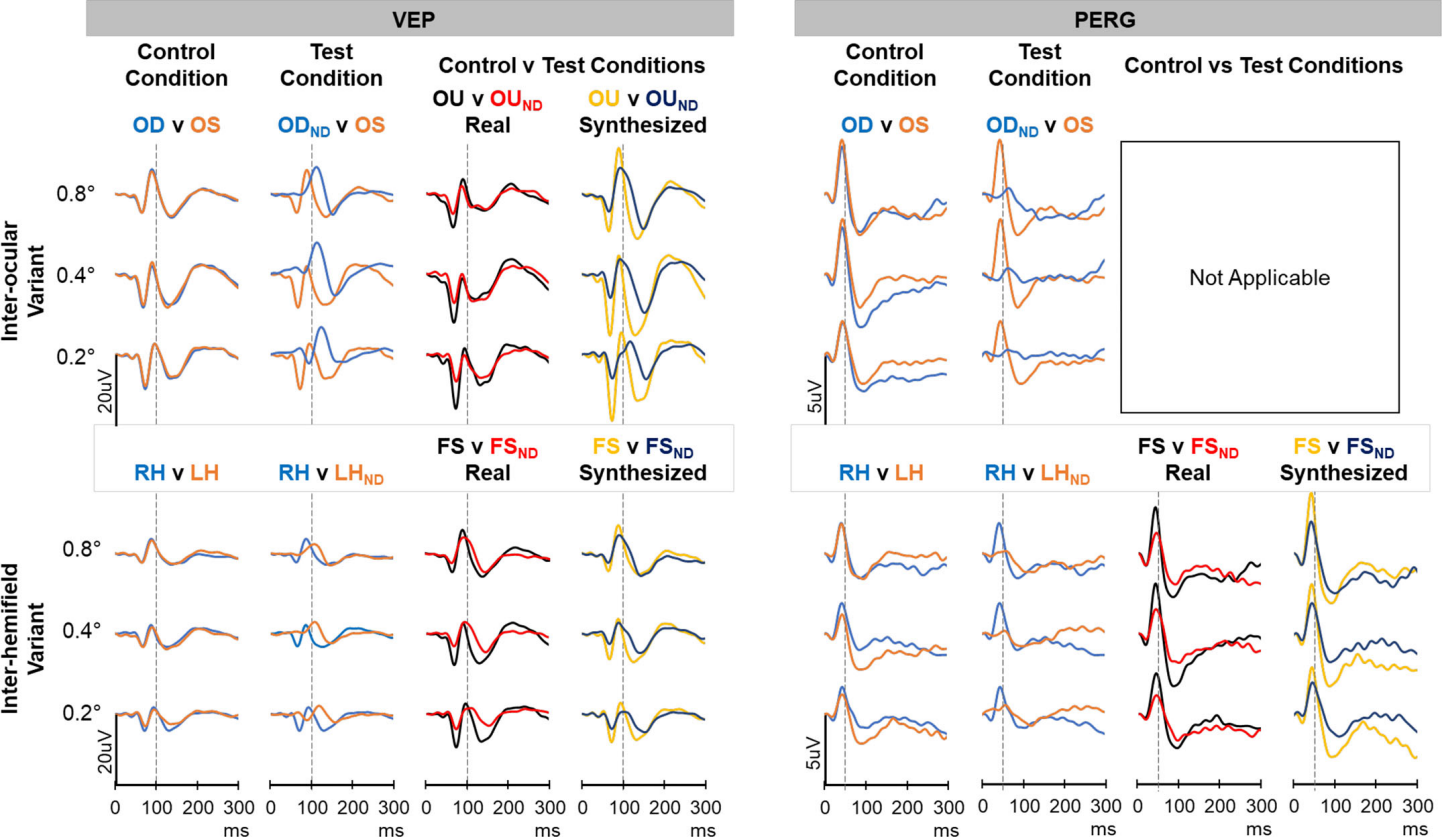
Grand mean traces for the various control and test conditions for three check sizes. The VEP traces are shown on the left half are composed of data from all the 15 participants whereas the PERG traces in the right half are composed of data from six participants.

The grand mean VEP and grand mean PERG traces for the participants for the 3 check sizes are shown in Figure 6. VEP traces are displayed in the left half whereas the PERG traces are on the right. The mean VEP amplitudes and peak times for all three check sizes for the various components are shown in Supplementary Table S1 for the interocular variant and Supplementary Table S2 for the inter-hemifield variant. Similar data for the PERG are shown in Supplementary Tables S3 and S4.

In summary, VEPs from the right and left eye in the interocular variant were very similar to each other for all three check sizes (column 1). This was also true for the VEPs from the right and left hemifield (RH versus LH) of the inter-hemifield variant. VEPs from the filtered eye (OD_ND_, interocular comparison) and the filtered hemifield (LH_ND_, inter-hemifield variant) were reduced in amplitude and delayed in time in comparison with their respective control non-filtered counterpart (column 2).

**Figure 7.**
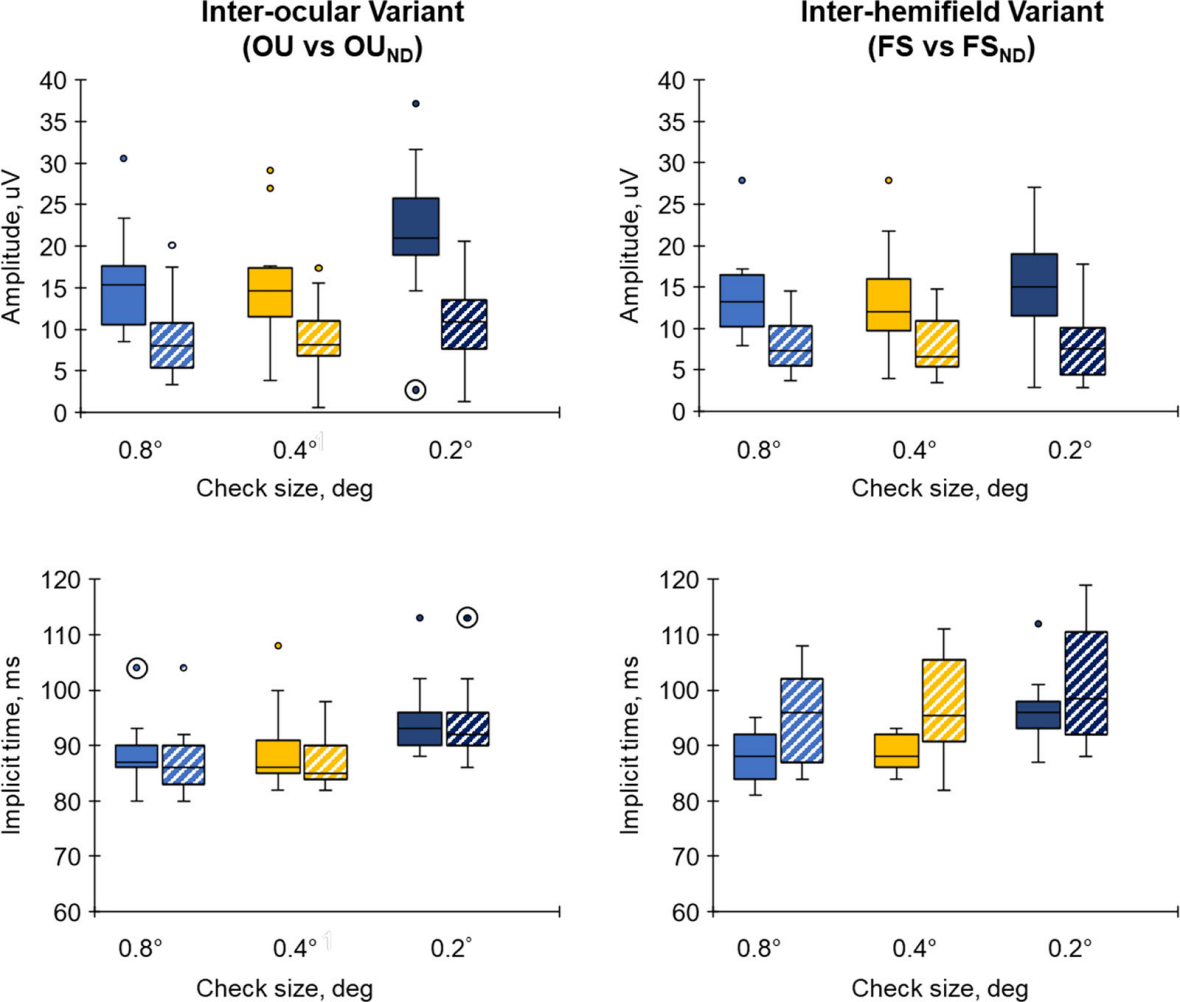
Comparison of P100 amplitude (*top row*) and implicit time (*bottom row*) between control (*solid colors*) and test (*hatched colors*) conditions for interocular (*left*) and inter-hemifield (*right*) conditions. Outlier data were values greater than 3 times the interquartile range above the third quartile (upper outliers) or below the first quartile (lower outliers) and are *circled*.

The binocular test condition (OU_ND_) of the interocular variant had a similar waveform to its counterpart binocular control condition (OU) for the various check sizes (column 3). The main observable differences were the reductions in the N75 and P100 amplitudes of the OU_ND_ in comparison with the OU. Data for the P100 are highlighted in Figure 7 (left column). There were statistical differences in mean amplitude between OU and OU_ND_ for all check sizes (*P* values < 0.001 for all check sizes). However, there were no statistical differences in the mean peak time between the OU and OU_ND_ (*P* values for 0.8 degrees, 0.4 degrees, and 0.2 degrees were for 0.902, 0.540, and 0.451, respectively). The comparison between the synthesized binocular VEPs for the test and control conditions (column 4) showed differences in the waveforms which were different to those observed in the comparison of the real binocular VEPs.

For the inter-hemifield variant, the waveform of the real monocular test condition (FS_ND_) was different to its control counterpart (FS) and the differences were most observable in the VEP for the 0.2 degrees check size. Notably the P100 peak of the FS_ND_ was broader, while the N135 was shallower and delayed in comparison with the FS. These are highlighted in Figure 7 for the P100 (right column). There were statistical differences in mean amplitude between FS and FS_ND_ for all check sizes (*P* < 0.001 for all check sizes). There were also statistical differences in the mean peak time between the FS and FS_ND_ for the 0.8 degrees (*P* = 0.005) and 0.4 degrees (*P* = 0.002) check sizes but not for the 0.2 degrees check size (*P* = 0.051). Furthermore, there was a larger intersubject variability in the FS_ND_ data compared to the FS data leading to considerable overlaps in the data especially for the 0.8 degrees and 0.2 degrees check sizes (see Fig. 7, bottom right). The comparison of the synthesized FS_ND_ and FS waveforms had similar features to those seen in the comparison of the real VEPs.

The PERG data (available for 6 participants), followed a similar pattern to the VEP with a few exceptions. For example, the PERG showed reduced amplitudes of the P50 and N95 components across the various check sizes for both interocular and inter-hemifield variants as seen in the VEPs. However, the amplitude reductions in the PERG were more pronounced than those in the VEP. The average percentage reduction in the P50 amplitude of the filtered eye in comparison with the unfiltered eye was 78%, 72%, and 70% for the 0.8 degrees, 0.4 degrees, and 0.2 degrees check sizes, respectively. The reduction in the N95 amplitude was 74%, 82%, and 79% for the 0.8 degrees, 0.4 degrees, and 0.2 degrees check sizes, respectively. Comparatively, the average percentage reduction of the P100 in the interocular comparison was 18%, 19%, and 21%, respectively, for the 0.8 degrees, 0.4 degrees, and 0.2 degrees checks.

The average delay in the P50 of the PERG of the filtered eye for the 0.8 degrees, 0.4 degrees, and 0.2 degrees check sizes were 22.5 ± 2.8 ms, 19.1 ± 8.2 ms, and 16.7 ± 8.0 ms, respectively; that for the N95 was 17.7 ± 6.9 ms, 13.0 ± 9.6 ms, and 10.4 ± 5.3 ms, respectively. These were comparatively lower than the average delays for the VEPs of the same six participants (i.e. 27.8 ± 5.8 ms, 26.8 ± 4.3 ms, and 30.2 ± 6.9 ms for 0.8 degrees, 0.4 degrees, and 0.2 degrees checks, respectively). For the inter-hemifield comparisons, the average delays in P50 of the filtered hemifield (LH_ND_) the 0.8 degrees, 0.4 degrees, and 0.2 degrees were 14 ± 7 ms, 9.7 ± 8.1 ms, and 15.7 ± 11.1 ms, respectively, whereas that for the N95 were 15 ± 9 ms, 13.0 ± 1.7 ms, and 29.0 ± 8.6 ms, respectively. These were comparable to the average VEP data of 19.5 ± 4.5 ms, 23.3 ± 4.7 ms, and 24.6 ± 7.7 ms for 0.8 degrees, 0.4 degrees, and 0.2 degrees checks, respectively. In spite of these delays seen in the averaged VEP and PERG data, there were no statistical correlations when individual data were compared.

## Discussion

This study examined the effect of conduction delays on the binocular and monocular pattern reversal VEP. The conduction delays were simulated in healthy individuals by creating a luminance difference between the two eyes (interocular variant) for the binocular VEP and creating a luminance difference between two hemifields (inter-hemifield) for the monocular VEP. Both test conditions were designed to produce a delay in approximately half of the signals arriving at the visual cortex.

The P100 is a prominent peak that shows relatively little variation between healthy subjects (<15 ms),^5^^,^^6^ minimal within-subject interocular difference (<12 ms),^22^^,^^23^ and minimal variation with repeated measurements over time (slows about 1 ms per decade from 5 to 60 years).^24^^,^^25^ In this study, the control VEPs from the right and left eyes, as well as from the right and left hemifields were very similar to each other (see Fig. 3) and were consistent with findings in previous studies.^22^^–^^24^^,^^26^^–^^29^ The 1.8 ND filter used in this study adequately altered the VEP of the filtered eye as it produced a 20% to 30% reduction in the P100 amplitude and a 20 to 30 ms delay between the filtered and unfiltered eye or hemifield. In the subset of participant in which PERG was recorded, the 1.8 ND filter reduced both the P50 and the N95 substantially (at least 70%). These are typical findings in optic neuritis^4^^,^^29^ and in other studies which have used similar neutral density filters of similar strength.^16^^,^^30^

For practical reasons, the present experiments were performed without dilation of the pupils. Although the reduction in retinal illuminance induced by the ND filter triggers a counteracting increase in pupil diameter, there is a net decrease in retinal illuminance as evident from the known Pulfrich effect and other sources,^31^ as well as from the fact that we see an effect in our data. However, we cannot exclude that some of the interindividual variability in our study originates from differences in iris pigmentation, which affects the dependence of overall retinal illuminance on pupil diameter,^32^ and retinal image contrast.^33^ This was not systematically assessed in the present study.

### Inter-Ocular Variant: Binocular VEP Produced by Luminance Difference Between the Eyes

The concept of this VEP experiment resembles that used to elicit the classical Pulfrich effect. Consistent with the assumption of a conduction delay underlying the Pulfrich effect,^13^ we found that reducing the luminance increased the peak time of the P100. With respect to the second experiment, this provided corroboration of our general experimental approach.

In the comparison between real VEPs of the test condition (OU_ND_) and its control (OU), the most noticeable differences were the reduction in the amplitudes of the N75 and P100 (see Fig. 6). Except for the reduction in the N75 and P100 amplitudes, the waveform shapes were very similar and there were no significant differences in the peak times of the three major components between the OU and OU_ND_. This occurred despite the significant delay in the timings of the N75, P100, and N135 of the filtered eye (OD_ND_) in comparison with the left eye (OS). This was consistent across all three check sizes (see Fig. 6). This indicated that the binocular VEPs, OU, and OU_ND_, were not the arithmetic sum of their respective component monocular VEPs, which is line with other studies.^22^^,^^26^^,^^27^^,^^34^^,^^35^ This was further seen in the discrepancy between the real VEPs and the synthesized counterparts (see Fig. 3).

The mechanism for this discrepancy is not fully understood, however, some authors suggest that there are separate cortical generators for the monocular and binocular VEPs,^34^^,^^36^ whereas others suggest nonlinear interactions between monocular cortical cells that would result in the activation under binocular conditions being reduced relative to the sum of monocular activations.^35^

### Inter-Hemifield Variant: Monocular VEP From Luminance Difference Between the Hemifields

In the comparison between real VEPs of the test condition (FS_ND_) and its control (FS), there were significant reductions in the amplitudes of all three major components across the three check sizes (see Fig. 6). In addition, there was a significant delay in N135 and a less pointy peak of the P100 which altered the waveform shape. This was most noticeable in the 0.2 degrees check size. Overall, the combination of amplitude reduction and prolonged peak time approximates the situation in moderate to severe acute optic neuritis, before recovery of the VEP amplitude.^4^

Despite the significant inter-hemifield delays between the RH and the LH_ND_, double peaks were not consistently observed in the FS_ND_ test condition of participants. Furthermore, the inter-hemifield differences between the RH and LH_ND_ peak time did not correlate with peak time of the FS for participants (see Fig. 4). Nevertheless, there was an observable tendency of an increasingly broadening P100 peak of the FS_ND_ with increasing inter-hemifield peak time differences. As noted in the introduction, this may be due to temporal dispersion.^7^^,^^8^ A related reasonable explanation is that conduction delays needed to occur in more than half of the axons to compensate for the effect of amplitude reduction that is normally present in the delayed component that produces double peaks in the VEP P100 in optic neuritis. It is also plausible that an increase in the inter-hemifield peak time beyond what was observed in this study (i.e. 29 ms) could have produced a double peak.

**Figure 8.**
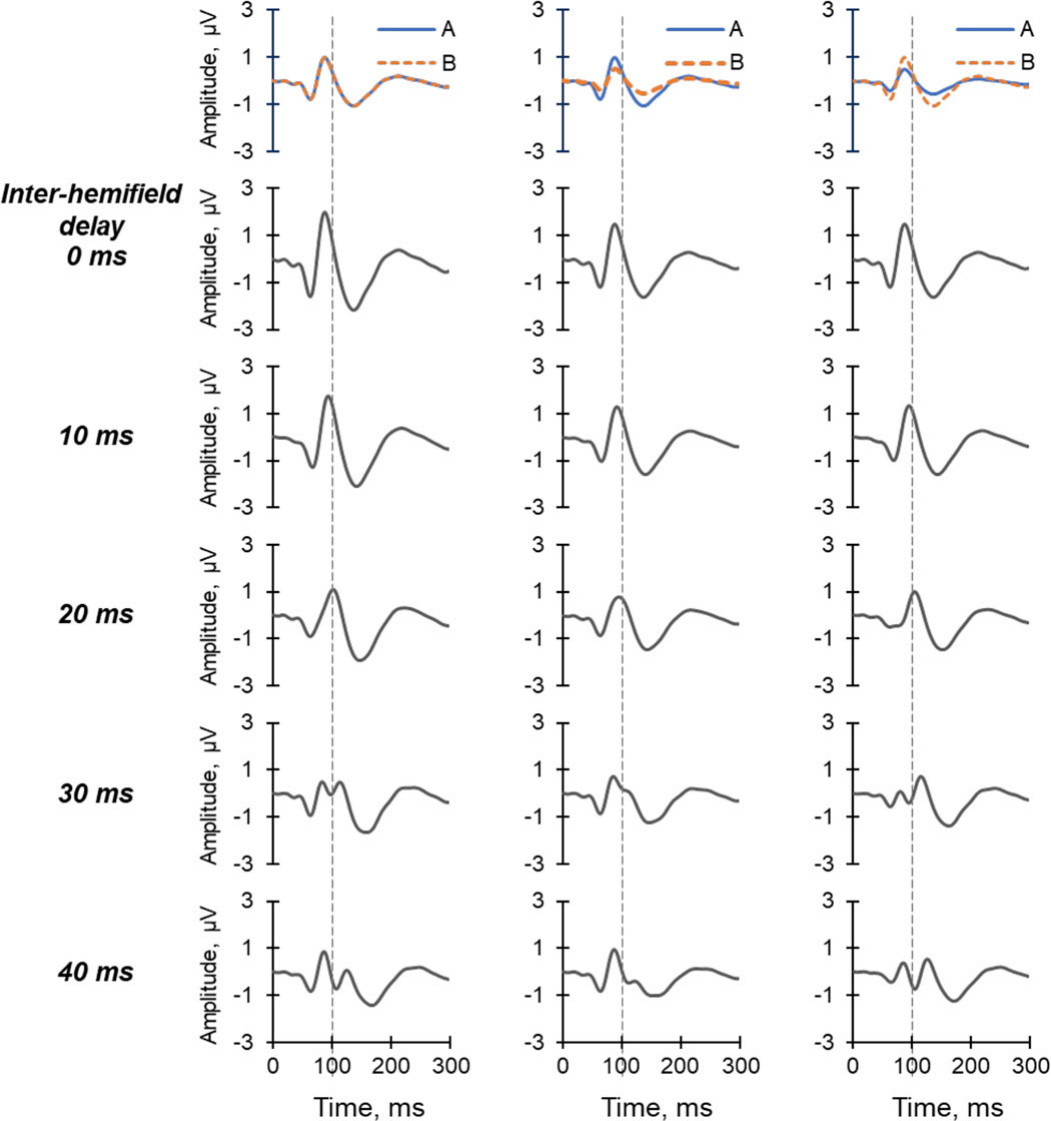
Interaction of hypothetical waves. The *first column* shows the results of combining two identical waves (**A** and **B**; *top row*) with increasing time differences in implicit time from 0 m to 40 ms in 10 ms steps (rows 2 to 5). The *second column* shows the case when wave A is twice the size of wave B and the final column shows the case when wave A is half the size of wave B. In all three cases, wave A is fixed in time, whereas wave B is shifted in time to the right.

To further examine these observations from the inter-hemifield variant, the effects of combining two hypothetical waves with different amplitudes and/or different time delays were modelled and are illustrated in Figure 8. When the two component waves are in alignment (0 ms delay), the resultant waves are simply the arithmetic sums of the component waves; equivalent to the complete summation described by Apkarian and colleagues.^34^ Apkarian and colleagues provided the following definitions for binocular interactions: full summation in binocular VEPs results if the amplitude of the binocular VEP (BVEP) is equal to twice the mean of the monocular responses (2*MVEP). If BVEP > 2*MVEP, this is known as facilitation, whereas if MVEP < BVEP < 2MVEP, then it is a partial summation. If BVEP < 1, then it is known as inhibition. Another situation which may result is suppression, in which BVEP is equal to the amplitude of the VEP with the higher amplitude.

When the time difference was increased to 10 ms and then to 20 ms, the amplitudes of the resultant waves showed partial summation and the peak time of the P100 increased. However, at a time difference of 30 ms and 40 ms, double P100 peaks emerged and there were ambiguities in which peak to consider for measuring the peak time of the P100. The observations from these hypothetical scenarios in Figure 8 gave credence to the assumption that double P100 peaks were more likely to occur when the inter-hemifield difference was 30 ms or more. Furthermore, they showed that the peak time of the resultant waves did not increase monotonically with an increase in the difference between the peak times of the component waves.

Our findings also showed that the sum of the hemifield VEPs (synthesized VEPs) for the test and control conditions closely matched their real counterparts. This corresponds to earlier studies which showed that the monocular VEP, which represents central retina activity, is the sum of VEPs from different parts of the central retina from which the monocular VEP was recorded.^37^^,^^38^ This is compatible with the idea that signal losses in the monocular VEP are proportional to the area of retinal loss within the central retina. However, the amount of signal loss per unit area may depend on the retinal location from which the signal was lost, as evidenced by the different check size dependence of the loss (see Fig. 7, top right graph). Furthermore, as the VEP primarily reflects the response to stimulation of the central visual field^1^ it is unclear from the present data whether spatial additivity generalizes to higher eccentricities.

In practical terms, the present data suggests that a comprehensive assessment of VEP recordings needs to include an evaluation of curve morphology in addition to simple peak time and amplitude readings, to avoid missing evidence of disease. Future studies will have to demonstrate how this can be achieved in a systematic, unambiguous, manner.

The small number of observations for the PERG (*n* = 6) precluded robust comparisons between the PERG and VEP data. Nevertheless, the amplitude reduction and peak time delay of the PERG in the filtered eye (OD_ND_) and hemifield (LH_ND_) were similar to observations made in the PERG in previous studies after luminance attenuation.^39^ This suggests that a sizeable fraction of the VEP peak time changes observed in the present study using a dimming technique originated in the retina. This contrasts with actual optic neuritis, in which the primary locus is the optic nerve, although effects on the PERG are sometimes observed in acute or severe cases.^29^^,^^40^^–^^42^

## Conclusions

The present findings corroborate the hypothesis that nominal peak time does not always reflect conduction delays if only a subset of fiber bundles is affected. P100 morphology is the result of interactions between changes in amplitude and peak time and can, for instance, remain completely unremarkable, be broadened, or shifted in time, exhibit a shoulder, or show a double peak. This implies that the rating of a VEP as “normal” (i.e. nonindicative of a disease such as optic neuritis) should not solely be based on the timing of the maximum of the largest peak.

## Supplementary Material

Supplement 1
