# Supplementary material for: Simulating the Effects of Partial Neural Conduction Delays in the Visual Evoked Potential
Source: Transl Vis Sci Technol. 2024 Feb 22;13(2):18. doi: 10.1167/tvst.13.2.18 (PMC10896232; doi:10.1167/tvst.13.2.18)
Supplement: Supplement 1 [file tvst-13-2-18_s001.pdf]

Supplementary Tables

Table S1. Amplitude and implicit time of the VEP components for the various recording conditions (horizontal) and check sizes (vertical) in the classical Pulfrich paradigm (Inter-ocular variant).

	Mean (OD and OS)			OU						OD_{ND}			OU_{ND}					
				Real			Synthesized						Real			Synthesized		
**Check size**	N75 µV	P100 µV	N135 µV	N75 µV	P100 µV	N135 µV	N75 µV	P100 µV	N135 µV	N75 µV	P100 µV	N135 µV	N75 µV	P100 µV	N135 µV	N75 µV	P100 µV	N135 µV
**0.8°**	**6.0**	**13.3**	**16.0**	**10.1**	**15.5**	**12.9**	**11.8**	**26.3**	**31.7**	**1.5**	**10.9**	**17.3**	**6.1**	**9.0**	**10.6**	**5.6**	**15.0**	**22.4**
±SD	2.6	5.0	6.0	5.0	5.8	6.6	5.3	9.9	12.1	2.1	3.7	5.7	4.1	4.8	4.9	3.2	5.8	8.5
**0.4°**	**8.9**	**12.8**	**13.9**	**13.6**	**15.0**	**10.4**	**17.0**	**24.3**	**26.1**	**0.9**	**10.4**	**17.0**	**8.3**	**9.1**	**10.1**	**7.6**	**13.6**	**17.8**
±SD	4.4	6.7	6.9	6.1	6.5	6.8	8.1	12.1	13.2	2.6	4.9	6.9	5.4	4.4	4.5	4.5	6.9	8.3
**0.2°**	**10.4**	**15.8**	**13.9**	**17.0**	**21.5**	**15.2**	**20.6**	**31.3**	**27.5**	**3.6**	**12.5**	**12.1**	**8.5**	**10.9**	**10.4**	**9.5**	**16.7**	**15.6**
±SD	3.0	6.0	6.3	5.4	7.9	7.9	5.9	12.0	12.8	3.4	3.8	3.1	4.6	5.0	6.2	2.7	6.0	6.8
**Check size**	N75 ms	P100 ms	N135 ms	N75 ms	P100 ms	N135 ms	N75 ms	P100 ms	N135 ms	N75 ms	P100 ms	N135 ms	N75 ms	P100 ms	N135 ms	N75 ms	P100 ms	N135 ms
**0.8°**	**64.3**	**88.1**	**138.2**	**64.2**	**88.4**	**135.1**	**64.1**	**88.2**	**137.5**	**71.5**	**115.0**	**154.5**	**64.4**	**87.3**	**132.2**	**64.8**	**100.5**	**151.6**
**±SD**	2.9	4.0	11.7	2.5	5.5	15.2	3.1	4.1	12.6	11.3	6.7	6.3	3.3	5.8	20.6	2.8	10.5	6.6
**0.4°**	**67.5**	**88.7**	**135.1**	**66.4**	**88.8**	**134.9**	**67.3**	**88.6**	**136.0**	**80.7**	**115.3**	**157.3**	**68.6**	**86.9**	**137.7**	**68.0**	**95.3**	**152.4**
±SD	2.2	3.1	11.4	2.5	6.9	14.2	2.2	3.2	13.1	7.3	7.2	7.8	5.9	4.2	16.9	3.27	11.02	7.05
**0.2°**	**73.6**	**96.6**	**142.0**	**72.1**	**94.4**	**137.5**	**73.3**	**96.7**	**140.4**	**92.0**	**126.0**	**160.9**	**73.4**	**94.0**	**139.5**	**76.6**	**107.9**	**158.2**
±SD	4.4	5.9	11.6	4.2	6.4	12.3	4.3	6.0	12.4	15.2	7.0	5.7	4.5	6.8	16.1	11.4	14.5	5.8

NB: Mean of OD and OS provided for simplicity due to high correlation and no significant differences between their means ($p < 0.001$). In view of the high correlation between the OD and OS, as well as between the RH and LH, only the mean values of the two eyes and the two hemifields are shown in the tables.

Table S2. Amplitude and implicit time of the VEP components for the various recording conditions (horizontal) and check sizes (vertical) in the modified Pulfrich paradigm (Inter-hemifield variant).

	Mean (RH and LH)			FS						LH_{ND}			FS_{ND}					
				Real			Synthesized						Real			Synthesized		
**Check size**	N75 µV	P100 µV	N135 µV	N75 µV	P100 µV	N135 µV	N75 µV	P100 µV	N135 µV	N75 µV	P100 µV	N135 µV	N75 µV	P100 µV	N135 µV	N75 µV	P100 µV	N135 µV
**0.8°**	**3.0**	**7.5**	**9.4**	**6.0**	**13.7**	**16.3**	**5.8**	**14.7**	**18.4**	**0.8**	**4.8**	**8.7**	**2.8**	**7.9**	**13.3**	**2.6**	**9.1**	**15.4**
±SD	1.8	2.7	2.8	2.6	5.0	6.7	3.5	5.2	5.3	1.3	2.2	2.8	1.6	3.3	5.4	1.9	4.4	5.8
**0.4°**	**4.1**	**6.8**	**7.9**	**9.3**	**13.2**	**14.4**	**7.8**	**13.0**	**15.0**	**1.1**	**5.3**	**8.2**	**3.7**	**7.5**	**10.1**	**3.7**	**7.9**	**11.7**
±SD	1.8	2.6	2.7	3.7	6.2	6.9	3.5	5.1	5.5	1.6	2.5	2.2	2.1	3.8	4.8	2.1	4.0	4.9
**0.2°**	**4.8**	**7.3**	**7.4**	**10.4**	**15.7**	**14.6**	**9.3**	**14.2**	**14.3**	**1.6**	**5.2**	**6.5**	**4.1**	**7.4**	**7.7**	**4.7**	**7.3**	**9.7**
±SD	1.6	2.8	3.3	3.9	6.2	7.3	3.3	5.7	6.6	1.7	1.9	2.0	2.8	4.4	3.8	2.6	3.2	3.6
**Check size**	N75 ms	P100 ms	N135 ms	N75 ms	P100 ms	N135 ms	N75 ms	P100 ms	N135 ms	N75 ms	P100 ms	N135 ms	N75 ms	P100 ms	N135 ms	N75 ms	P100 ms	N135 ms
**0.8°**	**63.9**	**87.5**	**140.3**	**63.8**	**88.0**	**139.5**	**63.7**	**88.9**	**140.3**	**67.1**	**108.8**	**149.5**	**63.0**	**94.7**	**144.4**	**62.3**	**94.7**	**146.9**
±SD	2.9	4.3	15.9	3.6	4.5	12.5	3.3	5.0	14.8	15.9	8.4	9.3	3.6	7.9	9.9	6.4	8.9	11.7
**0.4°**	**66.3**	**89.7**	**135.2**	**67.4**	**88.7**	**137.2**	**66.4**	**88.0**	**136.1**	**69.9**	**108.9**	**150.0**	**66.8**	**96.5**	**148.5**	**66.5**	**94.1**	**146.7**
±SD	3.7	8.2	10.9	2.3	3.2	14.1	3.4	3.5	14.4	16.7	9.5	12.2	3.5	8.8	7.5	5.7	11.7	11.4
**0.2°**	**72.0**	**95.6**	**142.6**	**72.9**	**96.2**	**141.5**	**73.1**	**93.9**	**141.2**	**83.8**	**120.0**	**156.1**	**72.1**	**101.1**	**151.6**	**71.7**	**105.5**	**152.3**
±SD	6.5	6.6	11.6	4.1	5.8	±11.4	+4.9	+6.2	13.4	9.2	13.7	8.9	2.8	10.2	10.1	10.0	12.5	12.1

In view of the high correlation between the OD and OS, as well as between the RH and LH, only the mean values of the two eyes and the two hemifields are shown in the tables.

Table S3. Amplitude and implicit time of the PERG components for the various recording conditions (horizontal) and check sizes (vertical) in the inter-ocular variant

	**Mean (OD and OS)**			**OD$_{ND}$**		
**Check size**	N35 µV	P50 µV	N95 µV	N35 µV	P50 µV	N95 µV
**0.8°**	**0.7**	**4.5**	**6.9**	**0.5**	**1.0**	**1.8**
±SD	0.5	1.9	1.9	0.4	0.8	1.5
**0.4°**	**0.7**	**4.5**	**7.3**	**0.7**	**1.3**	**1.3**
±SD	0.4	1.7	1.9	0.8	0.5	1.2
**0.2°**	**0.4**	**3.5**	**5.5**	**0.4**	**1.1**	**1.1**
±SD	0.4	1.7	1.4	0.9	0.3	0.6
**Check size**	N35 ms	P50 ms	N95 ms	N35 ms	P50 ms	N95 ms
**0.8°**	**18.3**	**42.2**	**91.8**	**36.7**	**64.7**	**109.5**
±SD	2.2	1.4	6.1	8.0	3.8	3.3
**0.4°**	**18.2**	**43.4**	**93.5**	**27.3**	**62.5**	**102.7**
±SD	1.2	2.2	10.0	10.5	8.0	9.1
**0.2°**	**18.8**	**44.2**	**95.8**	**27.2**	**60.8**	**106.2**
±SD	3.0	2.5	8.8	11.7	8.5	8.1

Table S4. Amplitude and implicit time of the PERG components for the various recording conditions (horizontal) and check sizes (vertical) in the inter-hemifield variant

	Mean (RH and LH)			FS						LH_{ND}			FS_{ND}					
				Real			Synthesized						Real			Synthesized		
**Check size**	N35 µV	P50 µV	N95 µV	N35 µV	P50 µV	N95 µV	N35 µV	P50 µV	N95 µV	N35 µV	P50 µV	N95 µV	N35 µV	P50 µV	N95 µV	N35 µV	P50 µV	N95 µV
**0.8°**	**0.44**	**2.53**	**4.00**	**0.89**	**4.16**	**6.33**	**0.85**	**5.03**	**7.90**	**0.33**	**0.84**	**1.60**	**0.65**	**2.25**	**3.34**	**0.76**	**3.26**	**5.00**
±SD	0.25	0.83	1.36	0.73	2.07	2.62	0.50	1.68	2.73	0.37	0.48	0.37	0.67	1.45	2.40	0.77	0.91	1.39
**0.4°**	**0.47**	**2.20**	**3.75**	**0.79**	**4.23**	**7.41**	**0.90**	**4.32**	**7.17**	**0.29**	**0.61**	**1.36**	**0.41**	**2.14**	**3.48**	**0.56**	**2.72**	**4.25**
±SD	0.15	0.92	1.70	0.57	1.74	1.90	0.27	1.87	3.55	0.49	0.40	0.45	0.49	1.34	2.28	0.58	1.03	1.75
**0.2°**	**0.24**	**2.06**	**3.22**	**0.40**	**3.15**	**5.37**	**0.41**	**3.95**	**6.24**	**-0.37**	**0.52**	**1.64**	**0.43**	**1.68**	**3.01**	**-0.09**	**2.63**	**4.06**
±SD	0.46	0.65	0.89	0.76	1.62	1.49	0.91	1.30	1.81	0.57	0.25	0.84	0.57	1.25	1.93	0.45	1.10	1.93
**Check size**	N35 ms	P50 ms	N95 ms	N35 ms	P50 ms	N95 ms	N35 ms	P50 ms	N95 ms	N35 ms	P50 ms	N95 ms	N35 ms	P50 ms	N95 ms	N35 ms	P50 ms	N95 ms
**0.8°**	**16.7**	**40.4**	**95.1**	**18.4**	**42.1**	**89.4**	**16.3**	**40.4**	**94.7**	**28.0**	**56.0**	**102.4**	**19.2**	**43.7**	**93.0**	**17.3**	**41.6**	**100.1**
±SD	1.3	1.6	5.2	3.4	1.0	4.8	1.2	1.9	6.8	10.2	6.6	9.9	3.7	4.6	12.7	1.5	3.0	10.4
**0.4°**	**17.9**	**42.6**	**90.5**	**17.7**	**42.6**	**95.9**	**17.7**	**42.4**	**88.7**	**28.3**	**53.7**	**95.6**	**19.2**	**45.0**	**90.5**	**18.3**	**42.1**	**89.3**
±SD	2.2	2.6	5.8	0.9	1.4	8.4	1.9	2.1	5.4	8.0	6.9	7.8	4.2	6.1	7.5	2.9	3.2	7.4
**0.2°**	**18.6**	**43.6**	**94.1**	**18.1**	**44.1**	**91.3**	**18.0**	**43.0**	**94.6**	**40.6**	**57.0**	**115.3**	**22.0**	**46.5**	**97.3**	**19.4**	**47.0**	**103.7**
±SD	1.2	2.3	9.9	2.2	2.4	9.5	1.9	2.0	10.1	6.9	10.6	4.0	7.3	5.6	5.2	3.3	8.4	9.6
